# Kneeling and standing up from a chair as performance-based tests to evaluate knee function in the high-flexion range: a randomized controlled trial comparing a conventional and a high-flexion TKA design

**DOI:** 10.1186/s12891-017-1657-3

**Published:** 2017-08-01

**Authors:** Paul J. P. van der Ven, Sebastiaan van de Groes, Jorrit Zelle, Sander Koëter, Gerjon Hannink, Nico Verdonschot

**Affiliations:** 1 611 Orthopaedic Research Laboratory, Department of Orthopaedics, Radboud University Medical Center, PO Box 9101, 6500HB Nijmegen, The Netherlands; 2 Department of Orthopaedics, Canisius-Wilhelmina Hospital, Nijmegen, The Netherlands; 3 Laboratory for Biomechanical Engineering, University of Twente, Enschede, The Netherlands

**Keywords:** Total knee arthroplasty, High-flexion, Performance-based tests, Functional outcome, Kneeling, Sit-to-stand

## Abstract

**Background:**

We compared the functional outcome between conventional and high-flexion total knee arthroplasty (TKA) using kneeling and sit-to-stand tests at 1 year post-operative. In addition, the patient’s daily functioning, pain and satisfaction were quantified using questionnaires.

**Methods:**

We randomly assigned 56 patients to receive either a conventional or a high-flexion TKA. Primary outcomes were maximum flexion angle and maximum thigh-calf contact measured during kneeling at 1 year post operatively. Secondary outcomes were the angular knee velocity and ground reaction force ratio measured during sit-to-stand performance tests, and questionnaires.

**Results:**

At one year post-operative, maximum knee flexion during kneeling was higher for the high-flexion TKA group (median 128.02° (range 108–146)) compared to the conventional TKA group (119.13° (range 72–135)) (*p* = 0.03). Maximum thigh-calf contact force was higher for the high flexion TKA group (median 17.82 N (range 2.98–114.64)) compared to the conventional TKA group (median 9.37 N (range 0.33–46.58))(*p* = 0.04). The sit-to-stand tests showed a significantly higher angular knee velocity in the conventional TKA group (12.12 rad/s (95%CI 0.34–23.91); *p* = 0.04). There were no significant differences between groups in ground reaction force ratios and patient-reported outcome scores.

**Conclusion:**

Although no differences were found in patient-reported outcome scores, differences in performance-based tests were clearly apparent. Standing up from a chair at 90° of knee flexion appeared to be easier for the conventional group. The kneeling test revealed significantly higher weight-bearing knee flexion for the high-flex group. Hence, if kneeling is an important activity for a patient a high-flex design may be recommendable.

**Trial registration:**

The study was retrospectively registered in ClinicalTrials.gov under identifier NCT00899041 (date of registration: May 11, 2009).

## Background

Several types of implant designs have been manufactured in order to optimize the results after total knee arthroplasty (TKA). Range of motion (ROM) is an important outcome parameter of postoperative knee function [1–3]. High-flexion designs are aimed at accommodating larger postoperative ROM necessary for activities of daily living (ADL), such as kneeling, standing up from a low chair, sitting cross-legged, transferring in and out of bath, gardening and stair climbing [4–9].

Design features of a high-flexion TKA are typically a reduced radius and an increased thickness of the posterior femoral condyle resulting in extended condyles. In addition, specific posterior-stabilized high flexion designs have an adapted post-cam mechanism providing increased femoral rollback [9–11]. However, it remains uncertain whether these design changes actually lead to functional benefits for TKA patients.

The results of TKA are mostly assessed using physical examination, X-rays and the evaluation of patient-based questionnaires. Although patient-based questionnaires provide feasible and appropriate methods to address the concerns of patients, they are subjective and assessment is often subject to floor or ceiling effects, which limits the adequate assessment of higher functioning patients [4, 5, 11, 12]. Moreover, most questionnaires were originally not designed for use in high-flexion TKA patients (e.g. no points were scored for extra ROM beyond 125°) [4, 5].

Performance-based testing, specifically targeted at high-flexion activities, has been suggested to help to compensate for the limitations in existing scores [4, 13]. One major advantage of performance-based testing is that pain and pain-related items do not have such a large effect on functional outcome as on patient-based questionnaires [1, 14–17].

Performance-based tests, such as sit-to-stand tests [16], and kneeling [18] have been proposed to evaluate knee function after TKA in the low-flexion (≤90°) and high-flexion range (>120°), respectively. However, during kneeling, thigh-calf contact has been reported to limit flexion and can therefore obscure the potential benefit reached with high-flex TKA designs [18, 19]. In that same study, thigh–calf contact pressures were shown to exponentially increase with increasing knee flexion angles, and to reach maximum values (up to >30%BW) in maximal flexion. Therefore, in order to assess TKA systems at high flexion, flexion angles as well as thigh-calf pressures need to be recorded.

In our clinic we traditionally use a PCL-retaining, fixed bearing device. However, high-flexion TKA systems may provide advantages for patients who perform high-demand activities (such as kneeling and sit-to-stand) on a daily basis. In order to determine whether a high-flexion TKA system would provide clinically relevant benefits for our patients we set up a randomized controlled trial to compare the functional outcome of our patients treated with either a PCL-retaining or a high-flexion TKA device.

Our primary objective was to compare the functional outcome between conventional and high-flexion TKA using kneeling as a performance-based test at one year post-operative. In addition, we compared the functional outcome between conventional and high-flexion TKA using a sit-to-stand test and we quantified patient’s daily functioning, pain and satisfaction using questionnaires at one year post-operative.

## Methods

We performed a prospective double-blind randomized controlled trial at the department of Orthopedics of the Canisius Wilhelmina Hospital, Nijmegen, the Netherlands. The study protocol was approved by the regional ethical committee (CMO 2008/021; ABR NL21274.091.08) and was carried out in line with the Helsinki Declaration. The study was retrospectively registered in ClinicalTrials.gov under identifier NCT00899041 (date of registration: May 11, 2009).

Patients with primary osteoarthritis or arthritis secondary to rheumatoid arthritis scheduled to undergo primary TKA were considered for inclusion and were enrolled prospectively. Exclusion criteria were: other causes of arthritis, inability to complete the exercises due to contralateral arthritis, contralateral TKA or other co-morbidities, and the inability to complete the questionnaires. Endpoints were defined as death, aseptic loosening, infection, amputation, reoperation or withdrawal on request.

In our protocol we explicitly specified any foreseeable post-randomisation exclusions; 1) death of the patient, 2) aseptic loosening of the prosthesis, 3) infection of the prosthesis, 4) amputation of the leg in which the prosthesis was placed, and 5) withdrawal on own request, as in these circumstances the outcomes of interest could not be measured.

**Fig. 1:**
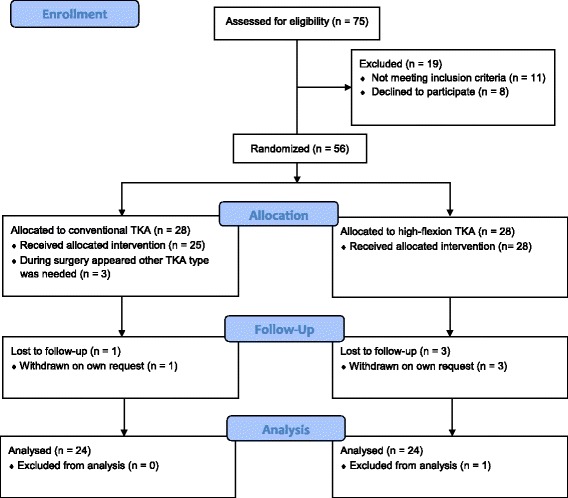
CONSORT 2010 flow diagram

Between November 2008 and November 2012, 75 consecutive patients undergoing unilateral TKA were assessed for eligibility (Fig. 1). Nineteen patients were excluded before randomization; eight patients declined to participate and 11 patients were excluded: mentally incompetent (1 patient), presence of contralateral TKA (2 patients), bilateral osteoarthritis (8 patients).

After written informed consent had been obtained, the patients were randomly allocated to receive either a PFC Sigma FB CR (fixed-bearing, cruciate-retaining; DePuy, Leeds, UK) or a PFC Sigma RP-F PS (rotating platform, posterior stabilized, high-flexion; DePuy, Leeds, UK). Computer-generated randomization with stratification for BMI below or above 30 kg/m^2^ was performed by an independent statistician. All patients and investigators were blinded for type of implant. The day before surgery the surgeon received a sealed study number envelope with the allocated TKA.

Identical surgical techniques were used in the groups according to the manuals of the designers. Three experienced knee surgeons were involved in this study. Rehabilitation was done according to the joint-care-protocol used in our hospital, including out of bed mobilization on the first postoperative day.

### Primary outcomes

The primary outcome measures were maximum flexion angle and maximum thigh-calf contact force measured during kneeling at one year post operatively. Maximal knee flexion angles during kneeling were measured using wireless accelerometers and gyroscopes (π-node, Philips, Eindhoven, the Netherlands). The accelerometers were positioned on the lateral side of both ankles, on both upper legs (10 cm above the patella) and on the sternum.

The maximal thigh-calf contact force (N) for the affected knee and unaffected knee were measured with a Conformat-pressure mapping sensor (Tekscan, Boston, USA). The pressure map was positioned in the popliteal fossa of both legs. The protocols for both measurements have been described in detail previously [18]. The mean of three consecutive maximum flexion angle and maximal thigh-calf contact force measurements was used in statistical analysis.

### Secondary outcomes

Sit-to-stand tests (STS) were used to assess the knee function in the flexion range up to 90° at one year post operatively. During STS we measured the angular knee velocity and ground reaction force ratio of both legs on the floor. The STS is a validated functional tool to assess knee patients which is selective and relatively independent of pain. The protocol has been described in detail previously [16, 20]. Angular velocity of the knee was measured using accelerometers, the ground reaction force (GRF) of each leg by two pressure plates [21]. TKA patients have been shown to produce a lower extension velocity while getting up from a chair as compared to healthy age-matched controls [15]. The ratio of ground reaction force (GRF_*ratio*_), which demonstrates the asymmetrical functional usage of the two legs, was expressed as the GRF of the TKA side (F_*TKA*_) divided by the GFR of the non TKA side (F_*no TKA*_): *GRF*_*ratio*_ *= F*_*TKA*_ */F*_*no TKA.*_

The patients’ daily functioning, pain and satisfaction were assessed using the following questionnaires: Knee Society Score (KSS), Western Ontario and McMaster Universities (WOMAC) and 0–100 Visual Analogue Scale (VAS) for pain and satisfaction (0 = no pain/extremely dissatisfied and 100 = very painful/very satisfied).

### Statistical analyses

Sample size estimation showed that 21 patients per group would be required to detect a clinically relevant difference of 10° of flexion with a standard deviation of 10° in knee flexion angle [10], with an alpha of 0.05 and a power of 90%. A dropout-margin of 7 patients for each group was used, which resulted in 28 patients per group. Descriptive statistics were used to summarize the data. Shapiro-Wilk tests and normality plots were used to assess normality. Differences between conventional and high-flex TKA designs were tested using Student t-tests and Mann-Whitney-U-tests for non-parametric and normal distributed data, respectively. With non-parametric tests, a measure of effect size, *r*, was calculated by dividing *Z* by the square root of *N* (*r* = *Z*/√*N*; small *r* ≥ 0.1, medium *r* ≥ 0.3, and large *r* ≥ 0.5) [22]. Analyses were performed using SPSS 21.0 (IBM, Chicago, USA). For all data sets, differences were considered statistically significant at *p*-values <0.05.

## Results

After randomization of 56 patients, three patients in the conventional TKA group were excluded: two because of insufficiency of the posterior cruciate ligament and one because an additional patella component was needed to improve patella tracking. During follow-up, one patient in the conventional TKA group was withdrawn on his/her own request without providing any reason. One patient in the high-flexion TKA group was withdrawn on his/her own request because of back problems. Two other patients in the high-flexion TKA group were withdrawn on their own request without providing any reason (Fig. 1). Patient demographics and baseline values are presented in Table 1.

**Table 1:** Patient demographics data and baseline clinical status

	Conventional TKA	High-flexion TKA
	(*n* = 24)	(*n* = 24)
Sex (F:M)^c^	11:13	12:12
Age (yrs)^a^	64 ± 7	66 ± 8
BMI (kg/m^2^)^a^	31 ± 4	32 ± 5
Thigh-calf contact force (N)^b^	15.88 (0–196.83)	9.70 (3.34–178.23)
Maximum flexion angle (°)^b^	127.7 (97–146)	126.6 (97–156)
Angular velocity (rad/s)^a^	80.56 ± 19.74	78.60 ± 18.26
GRF_*ratio*_ (1)^a^	0.86 ± 0.29	0.84 ± 0.26
KSS^b^	103 (55–132)	104 (78–151)
WOMAC^b^	55.5 (25–94)	49.5 (8–69)
VAS_*pain*_^b^	43.5 (0–90)	40 (0–99)

^a^Values are mean ± SD; ^b^Values are median (range); ^c^Values represent numbers

### Complications

In the conventional TKA group, one patient had a deep venous thrombosis treated with anti-coagulants 48 days post-operative, one patient had an inadequate knee flexion post-operatively and was treated with manipulation under anesthesia, and one patient had a patellar clunk and was treated using arthroscopic debridement. At 1 year post-operative, one patient in the high-flexion TKA group presented with signs of an infected TKA. Since an infected TKA was explicitly specified as reason for post-randomisation exclusion, and this patient was unable to perform kneeling and STS movements (and therefore no measurements could be obtained) this patient was excluded from the statistical assessment. However, later it appeared that all cultured biopsies were negative.

### Primary outcomes

#### Kneeling: Maximum knee flexion angle & maximum thigh-calf contact

At 1 year post-operative, maximum knee flexion during kneeling was higher for the high-flexion TKA group (median 128.02° (range 108–146°)) compared to the conventional TKA group (median 119.13° (range 72–135°)) (*U* = 174, *r* = 0.32, *p* = 0.03). Maximum thigh-calf contact force was higher for the high flexion TKA group (median 17.8 N (range 3.0–114.6 N)) than for the conventional TKA group (median 9.4 N (range 0.3–46.6 N)) (*U* = 177, *r* = 0.31, *p* = 0.04).

### Secondary outcomes

#### Sit-to-stand: Angular knee velocity & ground reaction force ratio

At 1 year post-operative, the angular velocity measured during sit-to-stand tests was higher for the conventional TKA group (93.23 rad/s (SD 21.94)) compared to the high-flexion TKA group (81.10 rad/s (SD 17.46)) (difference 12.12 rad/s (95%CI 0.34–23.91 rad/s); *p* = 0.04). No significant differences in GRF_*ratio*_ measurements between conventional (0.94 (SD 0.14)) and high-flexion TKA groups (0.87 (SD 0.21)) were found (difference 0.07 (95%CI -0.04 – 0.17); *p* = 0.21)).

### Questionnaires

At one year post-operative, no significant differences between conventional and high-flexion TKA groups in KSS, WOMAC, VAS_pain_, and VAS_satisfaction_ scores were found (Table 2).

**Table 2:** Results of primary and secondary outcomes

	Conventional TKA	High-flexion TKA	*p*-value
	(*n* = 24)	(*n* = 24)	
Thigh-calf contact force (N)^b^	9.37 (0.33–46.58)	17.82 (2.98–114.64)	0.04^d^
Maximum flexion angle (°)^b^	119.13 (72–135)	128.02 (108–146)	0.03^d^
Angular velocity (rad/s)^a^	93.23 ± 21.94	81.10 ± 17.46	0.04^c^
GRF_*ratio*_ (1)^a^	0.94 ± 0.14	0.87 ± 0.21	0.21^c^
KSS^b^	179 (90–199)	193 (109–201)	0.10^d^
WOMAC^b^	12.5 (2–62)	7 (0–54)	0.10^d^
VAS_*pain*_^b^	4 (0–54)	5 (0–31)	0.96^d^
VAS_*satisfaction*_^b^	89.5 (4–100)	98.5 (8–100)	0.06^d^

^a^Values are mean ± SD; ^b^Values are median (range); ^c^Student’s t-test; ^d^Mann–Whitney U test

## Discussion

In this study we compared the functional outcome between conventional and high-flexion TKA using performance-based tests at one year follow-up. It was found that during kneeling both the maximum flexion angle and thigh-calf contact force were significantly higher in the high-flexion TKA group. Sit-to-stand analyses showed no differences in asymmetry between the healthy and affected leg between conventional and high-flexion TKA group, while the patients in the conventional TKA group had a significantly higher angular velocity as compared to the high-flexion TKA group. Questionnaire scores (KSS, WOMAC and VAS scores) were similar in both groups.

Most previous clinical studies failed to show a difference between conventional TKA and high-flex TKA when using traditional outcome scores [4, 12, 13, 23, 24]. In addition, a recent study showed that current outcome measurement tools are not suited for the high flexion range [25].

In this study we found significant differences between conventional TKA and high flex TKA when using weight-bearing functional tests, but not when using traditional outcome scores proposed to evaluate knee function in the normal flexion range. The maximum knee flexion and thigh-calf contact forces during active kneeling were significantly higher in the high-flexion TKA group than in the conventional TKA group. The higher maximum thigh-calf contact in the high-flexion TKA group might be the result of the higher active flexion angle that was reached in that group. Since thigh-calf contact has been reported to limit flexion during kneeling, the flexion potential after high-flexion TKA might have been obscured by thigh-calf contact. In addition, although the surgeons used an identical surgical technique for both designs, it cannot be excluded that there were small differences in terms of treatment of the bone on the posterior region [9]. With the high-flex design more bone has to be removed at the posterior condyles, so it would be logical to also remove more posterior osteophytes and excessive bone that could possibly hamper high flexion. However, judging from the post-operative radiographs this could not be confirmed.

Remarkably, patients with a conventional TKA design produced a higher extension velocity during the sit-to-stand test. A higher angular velocity has been shown to be associated with a better functional performance [15]. Although a higher active flexion angle was obtained in the high-flexion TKA group, it apparently did not lead to a better performance of the extensor mechanism. Conflicting results between different post-operative outcome measures in the evaluation of high-flexion versus conventional TKA designs have also been reported by others [4, 12, 13, 23, 24, 26].

According to several authors performance-based measurements are necessary for an adequate evaluation of high-flexion TKA [4, 12, 13, 23, 24]. Nutton et al. [23] used performance-based measurements to evaluate functional outcome following TKA with NexGen standard and high flexion components. No significant differences in outcomes between patients receiving the conventional and high flexion designs were found. They divided performance-based measurements into ‘lower flexion’ and ‘higher flexion’ activities. The lower flexion activities were walking on a flat surface, ascending and descending a slope and a flight of stairs, and sitting and rising from a high chair. The higher flexion activities were sitting and rising from a low chair, getting in and out of a bath and bending the knee to the maximum range of flexion when standing, using a stool as a step. Finally, patients were asked to crouch and rise from a crouching position (squatting), using handrails for support. Patients were not asked to kneel, as most felt anxious about performing this activity. In addition, Palmer et al. [27] reported that some TKA patients were unwilling to kneel or squat because of advice from medical staff or third parties or because of fear of harming the prosthesis, although they state that no published data exists concerning this risk. The kneeling test used in the present study might therefore be a good method to distinguish between different TKA designs as the patient is in control of the movement.

The higher active flexion in the high-flexion TKA group is probably the result of the different design features and subsequent surgical aspects of the prosthesis. First positioning of the post-cam mechanism more posterior allows the knee to flex more due to a better rollback of the femoral component. Secondly, due to the thicker posterior condyles, high-flexion TKA surgery results in a better visualization of the posterior aspect of the knee allowing better decompression of posterior osteophytes and capsular tissue [4, 9]. Osteophyte removal could lead to a higher ROM in the high-flex range. Finally, adequate tensioning of the posterior cruciate ligament in the cruciate retaining prosthesis is challenging and the outcome is less predictable than in a posterior stabilized prosthesis and may have therefore jeopardized the ROM required for a kneeling exercise.

We did not find significant differences between conventional and high-flexion TKA when using the KSS, WOMAC and VAS scores. This is in line with previous observations reported by other authors [4, 7, 12, 23]. The self-reported questionnaires have a clear ceiling effect [4, 14, 17], and this makes them less useful for higher functioning TKA patients.

## Conclusion

This study showed that although no differences were found in patient-reported outcome scores, differences in performance-based tests were clearly apparent. Standing up from a chair at 90° of knee flexion appeared to be easier for the conventional group. The kneeling test revealed significantly higher weight-bearing knee flexion for the high-flex group. Hence, if kneeling is an important activity for a patient a high-flex design may be recommendable.

## Abbreviations

ADL: Activities of daily living
BW: Body weight
FB CR: Fixed-bearing, cruciate-retaining
GRF: Ground reaction force
KSS: Knee Society Score
PCL: Posterior cruciate ligament
ROM: Range of motion
RP-F PS: Rotating platform, posterior stabilized
VAS: Visual Analogue Scale
WOMAC: Western Ontario and McMaster Universities
